# Negative pressure wound therapy versus standard wound care in chronic diabetic foot wounds: study protocol for a randomized controlled trial

**DOI:** 10.1186/1745-6215-15-334

**Published:** 2014-08-27

**Authors:** Dörthe Seidel, Tim Mathes, Rolf Lefering, Martin Storck, Holger Lawall, Edmund A M Neugebauer

**Affiliations:** Institute for Research in Operative Medicine (IFOM), Faculty of Health - School of Medicine, University of Witten/Herdecke, Ostmerheimer Str. 200, Building 38, 51109 Cologne, Germany; Städtisches Klinikum Karlsruhe gGmbH, Moltkestr. 90, 76133 Karlsruhe, Germany; Asklepios Westklinikum Hamburg, Abteilung Angiologie/Diabetologie, Gefäßzentrum, Suurheid 20, 22559 Hamburg, Germany

**Keywords:** Diabetic foot wound, Amputation wound, Chronic wound, Foot ulcer, Diabetic foot, Foot diseases, Diabetes complications, Diabetes mellitus, Negative pressure wound therapy

## Abstract

**Background:**

In August 2010, the Federal Joint Committee (G-BA) decided that negative pressure wound therapy (NPWT) would not be reimbursable in German ambulatory care. This decision was based on reports from the Institute for Quality and Efficiency in Health Care (IQWiG), which concluded that there is no convincing evidence in favor of NPWT. The aim of this diabetic foot study (DiaFu study) is to evaluate whether the clinical, safety and economic results of NPWT are superior to the results of standard wound treatment.

**Methods/Design:**

The DiaFu study is designed as a national, multicenter, randomized controlled clinical superiority trial with a special focus on outpatient care in Germany. Competent patients in inpatient and outpatient care suffering from a chronic diabetic foot wound for a minimum of four weeks may be included in the study. The trial evaluates the treatment outcome of the application of a technical medical device which is based on the principle of NPWT (intervention group) in comparison to standard moist wound therapy (control group). All treatment systems used in the intervention group bear the symbol of free trade capacity in the European Union (CE mark) and will be operated within normal conditions of clinical routine and according to manufacturer’s instructions. Primary endpoints are the time to complete wound healing and the rate of wound healing achieved in each group within the maximum study treatment time of 16 weeks. Primary endpoints will be confirmed by blinded assessment of wound photographs.

**Discussion:**

The DiaFu study will provide solid evidence regarding the efficacy and effectiveness of NPWT until 31 December 2014, the date when G-BA plans to decide on future reimbursement of NPWT in both ambulatory and in-hospital care. The study is designed to comply with all quality requirements of G-BA and IQWiG and will contribute to evidence-based wound care in Germany. The study has been initiated by the statutory health insurance companies in Germany and is co-funded by two manufacturers of NPWT systems.

**Trial registration:**

Clinical Trials.gov registration number: NCT01480362 (date of registration: 23 November 2011).

German Clinical Trials Register number: DRKS00003347 (date of registration: 22 November 2011).

## Background

### Diabetes and diabetic foot wounds

Diabetes mellitus (DM) is a prevalent global chronic disease and describes a metabolic disorder. It is characterized by chronic hyperglycemia and disturbances in carbohydrate, fat and protein metabolism. These disturbances result from an inability of the pancreas to produce enough insulin, the body’s inability for an effective use of the produced hormone, or both.

The World Health Organization (WHO) estimates that more than 180 million people worldwide suffer from diabetes [1]. Even higher numbers have been predicted by the International Diabetes Federation (IDF), who reported in 2003 that 194 million adults were diagnosed with diabetes [2]. Both organizations estimate that in 2030 the number of those diagnosed will have almost doubled, mainly due to demographic changes [3]. Diabetes management involves a variety of costs and imposes enormous resource burdens, both on the individual and on healthcare systems. Diabetes and its sequelae cause significant economic consequences on individuals, families, health systems and countries. The WHO has estimated that 2.5 to 15% of annual national healthcare budgets are spent on diabetes-related illness alone [1].

The Diabetic foot wound is a common and frequent secondary syndrome that can lead to amputation. Due to arterial abnormalities and diabetic neuropathy, as well as a tendency to delay wound healing, infection or gangrene of the foot may develop. Acute and chronic wounds with healing impairment are a common problem of healthcare [4]. Complications associated with non-healing wounds range from inconvenient to life threatening and can be more common and serious than those related to the underlying disease. The occurrence of impaired wound-healing represents a multi-disciplinary treatment and cost-intensive clinical problem whether it is caused by infectious or non-infectious reasons.

### Modern wound treatment and negative pressure wound therapy

Modern wound treatment concepts include different types of moist dressings and topical agents, although only a few of these treatments have been convincingly shown to give higher wound closure rates compared with traditional wet gauze dressings [5, 6].

Negative pressure wound therapy (NPWT) was developed at the Wake Forest University (Winston-Salem, North Carolina) in the early 1990s [7, 8]. NPWT consists of an open-cell foam dressing covered with an adhesive drape. The dressing is connected to a vacuum pump that creates and maintains a sub-atmospheric pressure (intermittent or continuous). Positive effects of NPWT on wound healing have been demonstrated in basic studies [8, 9], and many case reports and case series document a broad use of NPWT in various clinical settings.

### Recent history of NPWT - clear evidence is still missing

In 2004 the German Federal Joint Committee (Gemeinsamer Bundesausschuss (G-BA)) commissioned an analysis of the available studies regarding NPWT to the Institute for Quality and Efficiency in Healthcare (Institut für Qualität und Wirtschaftlichkeit im Gesundheitswesen (IQWiG)) to support decision-making on the reimbursement of NPWT by the German statutory health insurance funds. The G-BA is the legislative institution of the German healthcare self-administration system. The IQWiG is an independent non-profit and non-government scientific institute that receives commissions from the Federal Joint Committee and the German Ministry of Health. The performed analysis aimed to evaluate the clinical benefit of NPWT in comparison with conventional forms of wound care, and to compare the benefit of various forms of NPWT for acute and chronic wounds with regard to patient-relevant outcomes on the basis of the published literature. The systematic examination of the clinical effectiveness and safety of NPWT compared with conventional wound therapy concluded that although there is some indication that NPWT may improve wound healing, the body of evidence available is insufficient to clearly prove an additional clinical benefit of NPWT. In addition, the large number of prematurely terminated and unpublished trials has been a reason for concern [10–12]. A 2007 Cochrane review concluded that trials comparing topical negative pressure (TNP) with alternative treatments for chronic wounds have methodological flaws and data do demonstrate a beneficial effect of TNP on wound healing. However, further better quality research is needed [13]. In 2011 Peinemann and Sauerland updated the systematic literature review [14] and concluded that although there may be a positive effect of NPWT, a clear evidence that wounds heal any better or worse with NPWT than with conventional treatment is still missing and better RCTs are still needed to evaluate NPWT. A Cochrane intervention review performed in 2013 concluded that there is some evidence to suggest that NPWT is more effective in healing post-operative foot wounds and ulcers of the foot in people with DM compared with moist wound dressings. However, these findings are uncertain due to the possible risk of bias in the original studies [15]. Therefore it can be concluded that current RCT evidence is limited and further trials are required to reduce uncertainty around decision-making regarding the use of NPWT to treat foot wounds in people with DM.

### Aim of the DiaFu study

This RCT is designed to prove that NPWT therapy leads more frequent and earlier to complete healing of diabetic foot wounds as compared to conventional moist wound therapy. Furthermore, it aims to show that NPWT therapy is also safe and effective when provided in an ambulatory care setting and to prove its overall cost-effectiveness. This trial is designed to comply with all quality requirements (randomized trial, low potential for bias; see Discussion section) of IQWiG and G-BA, as well as other European authorities.

## Methods/Design

**Figure 1:**
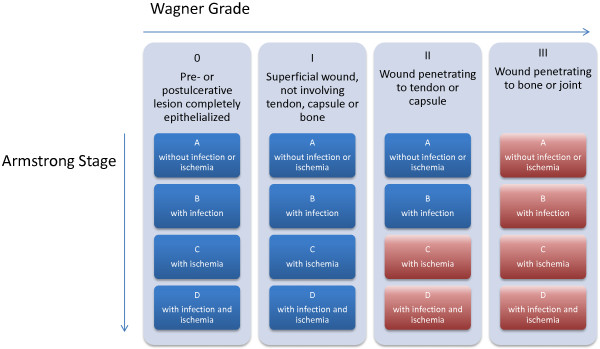
**Stratification of DiaFu study participants according to Wagner-Armstrong classification.** Participants of the DiaFu study are stratified by study center and by Wagner-Armstrong stage within each center (Group 1: <Wagner-Armstrong stage 2C and Group 2: ≥Wagner-Armstrong stage 2C). The table shows the modified Wagner-Armstrong classification scheme. Strata-Group 1 is marked blue and Strata-Group 2 is marked red.

The DiaFu study is designed as a national, multicenter, randomized controlled clinical superiority trial across Germany, with blinded photographic analysis of the primary endpoint. Participants are randomly allocated in a 1:1 ratio to each treatment group. Allocation to treatment groups is performed using a centralized web-based tool. To ensure good balance of participant characteristics in each group a stratified randomization is performed. Participants are stratified by study centre and by Wagner-Armstrong stage within each centre (<Wagner-Armstrong stage 2C and ≥ Wagner-Armstrong stage 2C; Figure 1). Due to the physical differences between the treatment regimens it is not possible to blind either participant or physician to the treatment arm. To address issues of blinding, confirmation of wound closure will be assessed via blinded assessment of wound photographs by independent observers.

### Phase and classification of the trial

The study is classified as an examination of the clinical application of CE-marked medical devices with risk category 2b (increased methodical risk) according to risk classification rules based on the annex IX of the European Union (EU) Directive 93/42 / European Economic Community (EEC). According to the German Medical Device Act (MPG) the trial is classified as an exemption to clinical investigation according to paragraph 23b MPG. This is due to the fact that the investigational devices are CE-marked, will only be operated within intended use and there will be no additional invasive or otherwise straining examinations. Examination results will only be documented if collected within clinical routine. Ethical approval of the Lead Ethical Committee (LEC, German: Federführende Ethikkommission) ‘Ethical Committee of the University of Witten/Herdecke’ has been fully granted without any conditions. As the trial will be performed in accordance with paragraph 23b MPG, further participating centers in Germany will only receive a professional legal advice for the respective main clinical investigator. Ethical approval of participating centers in Germany is not applicable.

### Setting

**Figure 2:**
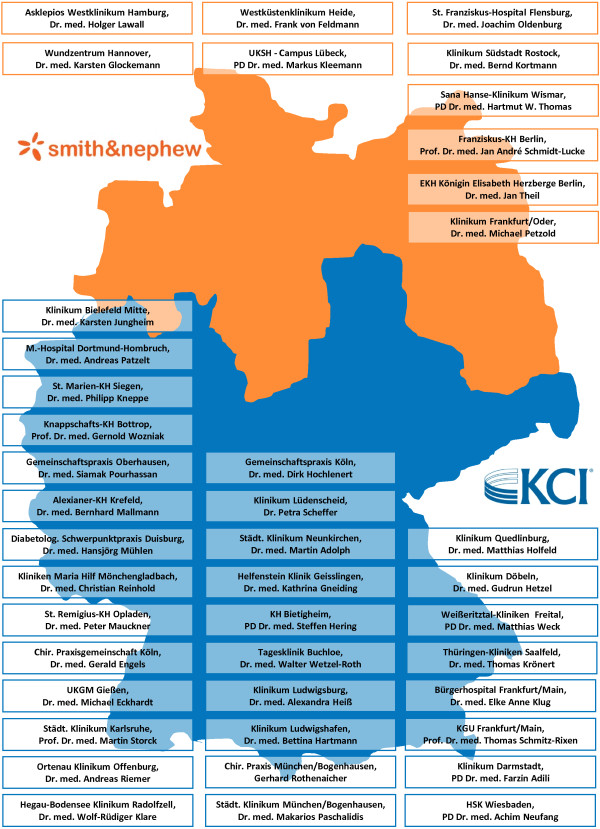
**DiaFu study sites.** Recruitment of study patients is performed in hospitals and outpatient facilities across Germany with a specific qualification for the treatment of chronic diabetic foot wounds. The table lists all study sites ready for patient recruitment as of 25 October 2013.

This multicentre study will be conducted in hospital departments and/or outpatient facilities with a special qualification for diabetic foot care (Figure 2). Study therapy will be started in-hospital or in ambulatory care and should be continued in ambulatory care whenever possible. The maximum study treatment time is set as 16 weeks after randomization and initiation of therapy. Randomized wound therapy must be started no later than six hours after the last wound preparation which can either be a wound cleansing, debridement or an amputation.

If a patient requires further local wound treatment after the end of the maximum study treatment time either standard wound treatment or NPWT may be performed as estimated by the treating physician to provide optimal wound care.

### Participants

**Figure 3:**
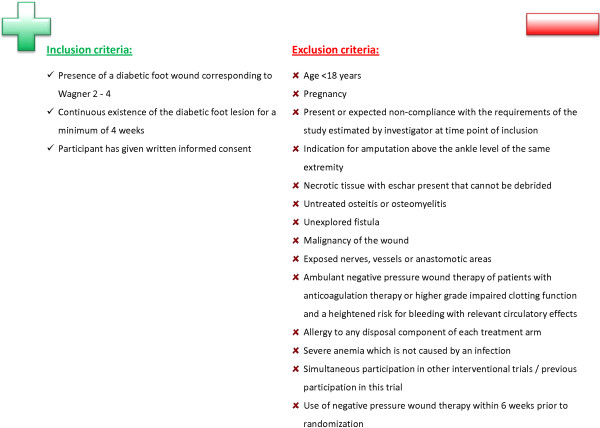
**Inclusion and exclusion criteria of the DiaFu study.** The table shows the required diagnosis and the inclusion and exclusion criteria for DiaFu participants.

All consecutive eligible adult patients (age >18 years) with a diabetic foot lesion corresponding to Wagner 2 to 4 that has continuously existed for a minimum of four weeks, who meet all inclusion criteria and no exclusion criteria, may be included in the trial. Chronic diabetic foot wounds after adequate wound pretreatment (debridement and/or wound cleansing) as well as amputation wounds resulting from a planned amputation underneath the upper ankle joint (lat.: Articulatio talocruralis) may be considered for inclusion. Therefore the main part of the wound is not allowed to be located above the upper ankle joint. Before randomization, inclusion in the trial and any trial-related procedure the participant must have provided written informed consent. Figure 3 provides a full overview of the inclusion and exclusion criteria. Reasons for non-enrolment or non-eligibility will be documented for all patients who fulfil the inclusion criteria but who are not included in the trial.

Any inability of the patient to respond to requests, to adequately assess risk or to comply with the requirements of the course of study, both in the inpatient and outpatient therapy episode, must be considered as non-compliance. If, in the estimation of the clinical investigator, a patient is non-compliant at the time of inclusion or is expected to be non-compliant during the course of the trial, this patient may not be included. Contraindications for the application of a NPWT device that result from a recommendation or a warning from the Food and Drug Administration (FDA) or from the manufacturers’ guidelines for product use are exclusion criteria for this trial. Patients with presence of necrotic tissue with slough and scab that cannot be debrided are not allowed to be included in the trial. Prior to randomization and start of study treatment, if necessary, a sufficient debridement or a radical removal of necrotic tissue in terms of an amputation should be carried out. If parts of the necrotic tissue cannot be removed by debridement or amputation, the patient should not be included in the study. Special attention has to be paid to exposed blood vessels within the wound or the direct wound surrounding that cannot be protected or that carry an increased risk of bleeding with relevant circulatory effects. If a sufficient covering is not possible, the patient should not be included in the study Patients may not be included if they are receiving anticoagulation therapy or are suffering from a higher grade impaired clotting function and have a heightened risk for bleeding with relevant circulatory effects if those patients are in outpatient care during the time of inclusion. If treatment systems which are based on the principle of NPWT have been used at the study-related foot lesion within a period of at least six weeks prior to the start of the study these patients may not be included in the trial. This is due to the objective of the study to demonstrate and evaluate a clear therapeutic effect of each treatment arm.

### Intervention and control

In the intervention group medical devices using the principle of NPWT will be applied on the study wound [7, 8]. This therapeutic technique uses a vacuum dressing to promote healing. The therapy involves the controlled application of sub-atmospheric pressure to the local wound environment, using a sealed wound dressing connected to a vacuum pump. NPWT promotes wound healing through optimization of blood flow, decreasing local tissue edema and removing excessive fluid from the wound bed. These physiologic changes facilitate the removal of bacteria from the wound. Additionally, the cyclical application of sub-atmospheric pressure alters the cytoskeleton of the cells in the wound bed through microstrain, triggering a cascade of intracellular signals that increase the rate of cell division and subsequent formation of granulation tissue. Moreover, this system has been shown to also cause macrostrain, bringing the edges of the wound closer and also stimulating wound bed granulation. NPWT as interim therapy should be discontinued once the condition of a wound is suitable for closing, either spontaneously or surgically.

Table 1 gives an overview of the NPWT devices used within the trial. Medical devices used within the trial are all CE certified products. All NPWT devices and dressings to be utilized in this study bear the CE mark and will be operated within normal conditions of use and according to clinical and manufacturers guidelines. Dressing changes are recommended to be performed according to manufacturer’s instructions with frequency adjusted by the clinician as appropriate. Contraindications and precautions will be noted.

**Table 1:** **Used medical products within the trial**

KCI	Smith & nephew
**NPWT devices**	
V.A.C. Freedom^®^	Renasys^TM^ GO
Acti V.A.C.^®^	Renasys^TM^ EZ Plus
INFO V.A.C.^®^	
V.A.C. Ulta^®^	
**Dressing**	
V.A.C^.®^ Granufoam^®^ (black)	Foam dressing (RENASYS^TM^ –F/P)
V.A.C^.®^ Granufoam^®^ silver^®^	Gauze dressing (RENASYS^TM^ –G)
V.A.C.^®^ Whitefoam^®^	

The table shows the NPWT devices of Kinetic Concepts Incorporated (KCI) and Smith & nephew used within the trial.

Control therapy is defined as any conventional moist wound therapy according to local clinical standards and guidelines [6]. Healthcare providers are obligated to provide patients with best practice and standard of care. In the control arm it is permitted to apply every local wound treatment standard used in the respective study site that does not have an experimental status or is based on the principle of NPWT.

To ensure the quality of local wound treatment during the course of the study, the study sites are trained for both the intervention arm and the control arm. The manufacturers are responsible for the training with the NPWT systems and consumables (dressing materials). The German Society for Wound Healing and Wound Treatment (German: Deutsche Gesellschaft für Wundheilung und Wundbehandlung e.V.) provides parts of their curriculum and performs the training for the control arm.

### Primary and secondary endpoints

Time until complete (100% epithelization) wound closure and the number of wound closures in each treatment group achieved in the maximum study treatment time of 16 weeks (112 days) are the primary endpoints of the DiaFu study. Complete wound closure is defined by 100% epithelialization of the wound (no granulation tissue visible), no drainage, no sewing material and no need for wound dressing or adjuvants. The assessment of complete wound closure is primarily based on the central blinded review of photographs performed by independent analyzers. Complete wound closure can be achieved either by surgery or by secondary intention. However, surgical intervention (for example wound suture, skin grafting or flap transplantation) is allowed only if the wound bed is prepared adequately. Sustainment of complete wound closure has to be proven by a satisfactory follow-up examination of the area 14 days after the intervention (wound closure verification visit). If the wound cannot be closed before the end of the maximum treatment time of 16 weeks, a study visit to assess the wound status and for evaluation of the secondary endpoints is performed.

Secondary clinical endpoints are the time until complete wound closure within the study time of six months, the number of wound closures per treatment group achieved within six months, the time until optimal preparation of the wound bed for further treatment (a minimum of 95% granulation), recurrences, amputations with incidence and extent, the wound size over time (area of wound opening, maximal length and width (cm^2^)) and the composition of wound tissue (percentage of epithelial layer, granulation tissue and fibrin).

The incidence of serious adverse events (SAEs) including mortality of any cause within six months from the time of initiation of therapy, incidence of device-related adverse events (ADEs) and incidence of wound-related adverse events (AEs) occurring within the study treatment time of 16 weeks or until wound closure confirmation are considered to be safety endpoints of this trial.

Furthermore, patient reported outcomes (PRO) will be evaluated. Quality of life (QoL) is measured using the questionnaire EQ5D. The questionnaire will be completed by the participant at inclusion, end of the maximum treatment time or end of the therapy and at the six-month follow-up visit . Furthermore, during study visits participants will be asked to provide their assessments of pain with a numerical rating scale (0 to 10). Participants will be asked to provide an estimate of their wound-associated pain of the last 24 hours at each trial visit.

### Health economic evaluation and analysis

In addition to the evaluation of clinical treatment effectiveness, a prospective assessment of health economic issues with a focus on the direct medical costs of wound treatment will be performed. This includes assessment of the parameters relevant for inpatient and outpatient resource use. A cost-effectiveness analysis using PRO and the number of wound closures as effectiveness measures will be performed. The economic evaluation is performed from the perspective of the German social insurance over the time horizon of the study period. To estimate costs the resource use is measured in natural units (for example duration of dressing changes in minutes). The average resource use per patient is calculated and priced with opportunity cost approximated over German market prices or schedule prices (for example wage agreements for staff costs). A sensitivity-analysis is performed by replacing the mean costs and effects with the upper and lower bound of the respective 95% confidence interval. Costs and effectiveness results are synthesized in an incremental cost-effectiveness ratio.

### Statistical analysis

The analysis of the primary and secondary endpoints will be performed according to the intention-to-treat principle (ITT). There will be no interim analysis. The ITT analysis population will include all randomized participants who have a valid baseline and at least one valid post baseline wound assessment (examination). For the ITT analysis population, participants will be assigned to the treatment group based on the randomization schedule, regardless of the treatment actually received. As a secondary approach a per protocol (PP) analysis will be performed excluding patients with any serious protocol deviations and changes of wound treatment. The rates of wound closure will be compared using Fisher’s exact test and time to complete closure will be compared using the Log-rank test. Safety data will be presented on an ‘as treated’ basis where patients will be grouped according to the exact wound treatment performed. Safety and secondary endpoints will be analyzed using conventional univariate testing. For the analysis of the primary efficacy endpoint missing values will be incorporated as censored values.

### Power calculation

The superiority hypothesis is tested in parallel with two different outcomes for the rate of wound closure and the time to wound closure in a fixed maximum treatment period of 16 weeks. The method of Bonferroni-Holm will be used for the adjustment of the α-error for parallel confirmatory testing of both primary endpoints. This means that after testing for both endpoints the smaller P value has to be <0.025 while the other has to be <0.05 for statistical significance. The sample size calculation is based primarily on the difference between wound closure rates in both treatment arms. The expected difference of the wound closure rates is based on information extracted from published previous studies. Armstrong and Lavery [16] described a rate of complete wound closure within 56% of patients with NPWT and within 39% of patients in the corresponding control group. Blume [17] showed a rate of complete wound closure within 43% of patients treated with NPWT and 29% of patients in the control group. For the present study success rates for achieving of the primary endpoint of 45% for NPWT and 30% in the control group are assumed, resulting in an absolute difference of at least 15% in the rate of complete wound closure results. Based on a type one error of α = 0.05 and a type two error of β = 0.2 (corresponding to a power of 80%) a total sample size of 162 patients per group (324 in total) is needed to prove the primary endpoint (wound closure rate).

## Discussion

### Trial preparation and organization

Within a European tender the study was initiated by the statutory health insurance in Germany. A consortium of 19 statutory health insurance funds including the Federal Association of Local Health Insurance Funds (AOK-Bundesverband), the Association of Substitute Health Funds (vdek-Verband der Ersatzkassen e.V.), and the Sickness Fund for Miners and Seamen (Knappschaft) including the Maritime Health Insurance Fund (See-Krankenkasse). These statutory health funds are the central contact for the study project and all associated health insurance funds provide integrated care contracts for ambulant NPWT of study patients. The Institute for Research in Operative Medicine (German: Institut für Forschung in der Operativen Medizin (IFOM) of the University of Witten/Herdecke is an independent scientific institute that is responsible for the scientific conception, analysis and reporting of the study. Furthermore, IFOM monitors the compliance with regulatory and quality requirements. The University of Witten/Herdecke has taken over the sponsorship of the trial. A management company, Gesundheitsforen Leipzig (GFL), is responsible for the logistics, the financial aspects and the study site selection, as well as patient recruitment for the study. The study database is provided and hosted by the GFL. The trial is funded by the participating statutory health insurance companies and the manufacturers Kinetic Concepts Incorporated (KCI) and Smith & Nephew (S&N). Both companies provide the NPWT systems in the assigned regions of Germany as well as all necessary information about the used medical devices and associated consumable supplies. Recruitment of study patients is performed in hospitals and outpatient facilities with a specific qualification for the treatment of chronic diabetic foot wounds all over Germany. A webpage provides more information about the overall study project. The website can be found using the uniform resource locator http://www.wound-care.de.

### Ensuring outpatient care of study participants with NPWT

In Germany outpatient care with NPWT is not generally reimbursed. To ensure the optimal patient-centered care and to avoid any bias, the participating health insurance companies are providing integrated care contracts for NPWT in the ambulant treatment sector. Moreover, integrated care contracts are provided for the ambulant NPWT of up to 7000 patients with acute and chronic wounds in Germany.

### Healing characteristics of chronic wounds and justification for the testing of two primary endpoints

Chronic diabetic foot wounds were chosen as the target study population because they are considered to be a fair representation of chronic wound types and also are frequently associated with the types of comorbidities observed with other types of open wounds. In chronic wounds the balance of individual physiological processes of wound healing is permanently disrupted and wound healing is extremely difficult or impossible. Regarding the healing time, stagnation within a non-healing state is observed. The treatment of chronic wounds is often characterized by prolonged or permanently insufficient applied therapeutic methods. The resumption of the physiological healing process in chronic wounds requires a longer start-up process than in the acute wound, where the physiological processes are in balance. The treatment of chronic wounds such as diabetic foot wounds is an interdisciplinary challenge. In addition to the burden on the patient, the associated health-economic costs can be enormous. According to Kaplan-Meier curves in the study of Blume [17] complete wound closure using NPWT was achieved after an average of 96 days, whereas the time for wound closure using standard therapy was not determinable. Thus, in chronic wounds the rate of achieved wound closures within a predetermined treatment time is of specific interest. This results in the definition of the primary endpoint as the number of wound closures within the maximum study treatment period of 16 weeks. In addition, a precise evaluation of the time required to reach a complete wound closure comparing the two arms of the study is of primary interest and of high economic importance.

### Definition of chronic wounds

The available literature on the definition of a chronic wound (for example expert standards and guidelines) shows a very heterogeneous picture [18] and ranges from four to eight weeks or more for minimum existence of the lesion. It was decided that patients who suffer for four weeks or longer from the continuous existence of a diabetes-associated foot lesion are suitable for participation in the present study.

### Wound closure

The FDA 2006 Guidance for Industry [19] defines complete wound closure as ‘skin closure without drainage or dressing requirements’. Wound closure can be achieved by delayed primary intention (secondary suture, skin flap or skin graft) or secondary intention according to requirements of the participant and wound in the estimation of the attending physician. Optimal care for the participant has to be ensured. Independent from type of closure, criteria for wound closure have to be reached within a maximum time frame of 112 days and wound closure has to be confirmed for a minimum of 14 consecutive days. Within each treatment arm surgical wound closure may only be performed if an adequate preparation of the wound bed (minimum 95% granulation tissue) has been achieved.

### Avoiding potential bias

Within the reports of the IQWiG and the updated systematic literature review of Peinemann and Sauerland [14] the risk of bias within the considered trials was examined using the predefined criteria shown in Table 2. All five criteria had to be met for the potential for bias to be described as low. The DiaFu- study is designed with the aim of avoiding all potential bias.

**Table 2:** **Potential bias**

Criteria for potential bias	Explanation
RCT	The performance of a randomized controlled trial is required.
Suitable allocation to groups	A precise description of the randomization sequence generating procedure is required (such as computer-generated lists).
Allocation to groups suitably concealed	Information on how allocation to groups is blinded is required (for example centrally by telephone or using sealed opaque envelopes).
Assessment of endpoints blinded	Information on who (patient and/or researcher) assessed which endpoint under blinding conditions (without knowing the group to which the patient had been allocated) is required.
Reasons given for any data loss	The requirement is either no data loss or, if data loss is reported, identification of all patients whose data could not be fully evaluated after randomization and the reasons for this (for example patients who dropped out before the beginning of treatment or during follow-up).
Adequate ITT analysis	Evaluation using the number of randomized patients as the size of the population is required

### Ethical conduct of the study

This study is conducted in accordance with the International Conference on Harmonization (ICH) Harmonized Tripartite Guidelines for Good Clinical Practice 1996, the European Union (EU) Directive 95/46/EC on the protection of individuals with regard to the processing of personal data and on the free movement of such data as transposed into national law, the EU Medical Device Directive 93/42/EC as amended by Directive 2007/47/EC as amended into national law, the International Organization for Standardization (ISO) 14155 related to AE definitions and in the spirit of the Declaration of Helsinki concerning medical research in humans (latest edition).

### Publication of study results

Both positive and negative results of the trial will be disclosed. The results of the trial will be submitted for publication to peer-reviewed scientific journals and presented at international scientific congresses. The Principal Investigator and the Steering Committee are responsible for the primary and secondary publications and/or presentations arising from the study. All publications will maintain data protection of participant data as well as data of the participating clinical investigators. Results will be reported according to CONSORT recommendations [20].

## Trial status

Recruiting since December 2011.

Stop of recruitment estimated for 10/2014.

## Authors’ information

Dörthe Seidel is a physician with a focus on surgical research and wound healing. She is the Principal Investigator (German: Leiterin der Klinischen Prüfung (LKP)) of the DiaFu study according to the German Medical Device Act. In the Centre for Clinical Trials and Innovation (German: Zentrum für Klinische Studien und Innovation (ZKSI)) (Department for Clinical Research) she is responsible for the medical and scientific issues.

Tim Mathes is a Dipl. health economist with a study focus on statistics and evidence based medicine. He works as research associate in the department for evidence-based health services research and the ZKSI. His main task is statistical, methodological and health economic consulting. He has experience in the preparation of systematic reviews and meta-analyses.

Professor Dr Rolf Lefering is a mathematician and very experienced statistician with a special focus on registry research. He is head of the department for biometrics and registry research.

Professor Dr med. Martin Storck is a professor at the University of Freiburg and the director of the department for vascular and thoracic surgery at the Klinikum Karlsruhe, a maximum care hospital. The department is involved in several clinical trials including FDA studies. He is also a board member of the German Society of Vascular Surgery.

Dr med. Holger Lawall is a vascular physician and diabetologist with particular scientific interest and work in the field of PAD, diabetic foot syndrome and critical limb ischemia. He is the director of the vascular centre at the Asklepios Westklinikum Hamburg.

Professor Dr Edmund AM Neugebauer is a professor and Chairman of Surgical Research at Witten/Herdecke University in Cologne. His is Dean for Research at the university’s Faculty of Health, School of Medicine and Director of the Institute for Research in Operative Medicine. He is also currently the President of the German Network for Health Services Research (DNVF).

## Abbreviations

ADEs: Adverse device events/device-related adverse events
AEs: Adverse events
EC: Ethical committee
EU: European Union
FDA: Food and Drug Administration
G-BA: German: Gemeinsamer Bundesausschuss (English: German Federal Joint Committee)
GFL: German: Gesundheitsforen Leipzig
ICH: International Conference on Harmonization
IDF: International Diabetes Federation
IFOM: German: Institut für Forschung in der Operativen Medizin (English: Institute for Research in Operative Medicine)
IQWiG: German: Institut für Qualität und Wirtschaftlichkeit im Gesundheitswesen (English: Institute for Quality and Efficiency in Healthcare)
ISO: International Organization for Standardization
ITT: Intention-to-treat
KCI: Kinetic Concepts Incorporated
LEC: Lead ethical committee (German: Federführende Ethikkommission)
MPG: German: Medizinproduktegesetz (German Medical Device Act)
NPWT: Negative pressure wound therapy
PP: Per protocol
PRO: Patient reported outcome
QoL: Quality of life
S&N: Smith & Nephew
SAEs: Serious adverse events
TNP: Topical negative pressure
WHO: World Health Organization.
